# Use of spirometry and recording of smoking habits of COPD patients increased in primary health care during national COPD programme

**DOI:** 10.1186/1471-2296-12-97

**Published:** 2011-09-21

**Authors:** Tuula Vasankari, Anne Pietinalho, Kalle Lertola, Seppo YT Junnila, Kari Liippo

**Affiliations:** 1 Finnish Lung Health Association, Sibeliuksenkatu 11 A 1, FI-00250 Helsinki Finland; 2 Department of Respiratory Medicine, Turku University Hospital, Paimio Hospital, Alvar Aallon tie 275, FI-21540 Preitilä Finland; 3 Turku University, FI-20014 Turun Yliopisto, Finland; 4 Salo Healthcare Center, Sairaalantie 9, FI-24130 Salo, Finland

**Keywords:** COPD, diagnosis, primary healthcare, respiratory symptoms, spirometry, smoking

## Abstract

**Background:**

In Finland, a national programme for COPD prevention and treatment was developed in 1998. The main goals of the programme were to diagnose COPD as early as possible and to encourage people to quit smoking. The role of primary health care was emphasized in the programme. Our aim was to investigate the use of spirometry and recording of smoking habits of COPD patients in primary health care before and during the COPD programme.

**Methods:**

We compared patients with respiratory symptoms or diseases visiting primary health care during 1997 (before programme) and 2002 (during programme). Patients with respiratory symptoms were divided into two groups: COPD patients and "others". Patient records were thoroughly investigated and data retrieved from them.

**Results:**

There was a significant increase in the whole study group from 8.0% to 38.9% in the use of spirometry (p < 0.001). This increase was significant both in the COPD group (from 32.0% to 79.6%, p < 0.001) and "others" (from 5.6% to 32.8%, p < 0.001). Written information on smoking habits in patient records increased from 16.6% of all patients in 1997 to 53.2% in 2002 (p < 0.001), and in COPD group from 45.0% to 84.3% (p < 0.001).

**Conclusions:**

We observed a significant increase in the use of spirometry and knowledge of smoking habits in COPD patients, which may be a result of the Finnish national COPD programme.

## Background

Chronic obstructive pulmonary disease (COPD) is a major health problem causing morbidity and mortality worldwide [1-3]. The incidence of COPD is increasing and its financial impact escalating [4]. International and national guidelines of COPD have been published recently to standardize in order to make the diagnosis and treatment of the disease equal in different settings [5-7].

COPD should be diagnosed using the appropriate measurements of lung function [8-11]. In the early stage of COPD airflow obstruction can be present without causing symptoms. Especially this group warrants an early diagnosis, and the role of primary healthcare physicians is crucial in this respect [9,12]. There are some reports that indicate shortcomings of the detection of COPD in primary care such as underuse of spirometry, in its diagnosis [13-16]. Cigarette smoking is the major risk factor for the disease, and the importance of smoking cessation has been shown in preventing further decline of lung function [17]. Knowledge of patients smoking habits is essential both in diagnosis and treatment of COPD [18].

In Finland, the National Programme for Chronic Bronchitis and Chronic Obstructive Pulmonary Disease 1998-2007 was designed by The Finnish Lung Health Association (Filha, NGO) in cooperation with the Ministry of Social Affairs and Health and the Pulmonary Association Heli (NGO) with the help of a working group of experts. It was subsequently published on behalf of the Ministry of Social Affairs and Health in 1998 for 10 years [19]. The main goals of the programme were to diagnose COPD as early as possible and to encourage people to quit smoking. The role of primary health care in the diagnosis and treatment of COPD was emphasized in the programme. A nation-wide implementation was performed in specialized as well as in primary health care, coordinated by Filha. At first, they were collaborating together with all [21] hospital districts (especially their pulmonary clinics) and all [6] occupational health districts to organize training events together with hospitals, and primary health care personnel were also invited. The topics were COPD as a disease, diagnosis of COPD (spirometry), smoking cessation and treatment of COPD. Secondly the training events were organized in collaboration with 156 (of 270) primary health care centers that were interested and willing to participate in this training. These sessions were arranged at health care stations during the working day (2-4 hours per time). All training was multidisciplinary. Approximately one third of the participants were doctors and two thirds nurses. From 1997 to 2002, approximately 210 training events for recognition of COPD were arranged around the country. As many as 8 200 health care professionals attended the meetings. Two information and training opportunities were offered to the personnel of each health care center during 5 years. The evidence-based Finnish Current Care Guidelines for COPD were introduced in 1999, and were also included in the implementation [20]. As far as we know there are no data concerning the effects of national programmes on diagnosis of COPD in primary health care.

The present study aimed to investigate the use of spirometry in diagnosis and follow-up of COPD patients and recording of smoking habits in a primary health care center before and during the national COPD programme.

## Methods

A medium-sized primary healthcare center in south-west Finland with computerized patient records was chosen for the study. The healthcare center chosen used electronic patient record system for all patient data archiving. For every patient visit an International Classification of Primary Care (ICPC) code was recorded, and information concerning the cause of visit, clinical findings, results of examinations and treatment suggested was documented. Subjects were all aged > 16 years to include all adults visiting primary health care due to respiratory symptoms. The year 1997 was chosen to represent the situation in the country before the introduction of the 1998 national programme and campaign for detecting and treating COPD, and the year 2002 to represent the impact of the programme.

The population living in the area of this healthcare center in 1997 was 44 402, of whom 36 170 were aged > 16 years. The figures for 2002 were 46 063 and 37 444, respectively. In 1997 and 2002, the number of doctor visits for all causes was 85,535 and 89,787, respectively. For every visit a main diagnosis routinely documented by the treating physician to the healthcare center records was available for the study. All the visits due to respiratory symptoms or diseases were included in the study. Visits due to COPD were identified retrospectively with the help of ICPC (International classification of primary care) [21] and International Statistical Classification of Diseases and Related Health Problem (ICD10) codes recorded. ICPC codes were used almost solely, and ICD10 codes were used only during primary health care in-hospital periods. In-hospital and polyclinical treatment in specialized health care were not included in the study. The doctors responsible for diagnosing and treating patients did not know about the study when they met the patients. Our aim was to discover all new and pre-diagnosed COPD patients visiting primary health care during each study year. We also included codes of other obstructive respiratory diseases and symptoms suggestive of COPD to find those COPD cases remaining undiagnosed. The overall diagnostic efforts done due to suggestive respiratory symptoms by general practitioners were studied. The diagnosis codes included were those of chronic bronchitis, emphysema, COPD, asthma, cough, dyspnea, wheezing, abnormal sputum and bronchiectasis.

Based on medical record data, patients with respiratory symptoms were divided into two groups: COPD patients and "others". The COPD group was of primary interest. All the available data, including those before our study period, were used.

To be included in the COPD group a patient had to have:

- An earlier diagnosis of COPD made in specialized health care (respiratory clinic).' or

- A suitable history and deteriorated spirometric values. FEV1 had to be ≤ 80% of the predicted value and the FEV1/FVC ratio ≤ 80% according to Finnish clinical practice and guidelines [19,22].

and

- Asthma excluded with peak expiratory (PEF) flow measurement data (those having repeatedly ≥ 20% of diurnal variation were excluded from the COPD group).

Other cases formed the group of "others" with respiratory symptoms. Some had asthma or other respiratory diseases, but most had only a variety of respiratory symptoms without specific diagnosis, or at least no diagnosis was made according to the data available.

Pack-years of smoking were computed for current and ex-smokers. One pack-year was regarded as equivalent to 20 cigarettes smoked daily for 1 year. The variables studied included sex, age, history of smoking, PEF and spirometric values.

The main outcomes of the study were the frequency of the use of spirometry for the diagnosis of respiratory symptoms suggestive of COPD and the adequate recording of smoking habits of those seeking medical advice due to their respiratory symptoms.

The statistical analysis was carried out using SPSS and SAS software. Categorical variables were tested by chi-squared test, numerical variables by analysis of variance.

The study was approved by the Salo Healthcare Center Ethical Committee and was performed in accordance with the principles of the Declaration of Helsinki.

## Results

In 1997 and 2002 a total of 1 072 and 1 645 patients with respiratory symptoms were included, respectively. The share of men was 38.3% (411) in 1997 and 37.0% (609) in 2002 (Table 1).

**Table 1:** Characteristics of the study patients, knowledge of their smoking habits, and use of spirometry in the study groups, years 1997 and 2002

	Group	1997	2002	p
N	COPD	100	216	NA^1^
	
	Others	972	1429	NA
	
	All	1072	1645	NA

Mean age (years ± SD)	COPD	70.1 (11.3)	68.3 (13.0)	0.22
	
	Others	51.7 (19.8)	53.4 (19.3)	0.03
	
	All	53.4	55.4	0.96

Gender/male N (%)	COPD	69 (69.0)	146 (67.6)	0.46
	
	Others	342 (35.2)	463 (32.4)	0.09
	
	All	411 (38.3)	609 (37.0)	0.49

PEF	COPD	173.0 (260.3)	309.3 (148.4)	0.001
	
	Others	263.3 (227.1)	414.8 (121.0)	< 0.001
	
	All	247.1 (235.0)	390.8 (135.0)	< 0.001

FEV1/FVC	COPD	61.9 (10.6)	67.4 (14.0)	0.04
	
	Others	81.8 (6.4)	86.1 (18.1)	0.10
	
	All	74.2 (12.7)	81.1 (19.0)	0.001

Data on smoking status available N (%)	COPD	45 (45.0)	182 (84.3)	< 0.001
	
	Others	133 (13.7)	693 (48.5)	< 0.001
	
	All	178 (16.6)	875 (53.2)	< 0.001

Pack years of smoking	COPD	31.0 (9.8)	46.9 (17.9)	0.03
	
	Others	25.9 (9.3)	24.5 (18.8)	0.86
	
	All	27.2 (11.5)	37.3 (19.2)	0.03

Spirometry performed N (%)	COPD	32 (32.0)	172 (79.6)	< 0.001
	
	Others	54 (5.6)	468 (32.8)	< 0.001
	
	All	86 (8.0)	640 (38.9)	< 0.001

^1 ^Not available

The diagnosis of COPD had been made in 100 cases in 1997 and in 216 cases in 2002, or medical record data made it possible to confirm COPD and exclude asthma by the study team. These cases were included in the COPD group. The remaining 972 patients in 1997 and 1 429 in 2002 formed the group "others". In 1997 332 (34.2%) of others had asthma, 7 (0.7%) had chronic bronchitis and the reminder, 633 (65.1%), of that group were without any specific diagnosis. The figures for 2002 were 443 (31.0%), 26 (1.8%) and 960 (67.2%) respectively. The demographic details of the groups are shown in Table 1.

Altogether 29.6 patients per 1 000 persons visited primary healthcare for respiratory symptoms in 1997, the respective figure was 43.9 per 1 000 in 2002. The number of patients visiting primary health care due to COPD was 2.4 per 1 000 persons in 1997 and 5.8 per 1 000 in 2002.

There was a significant increase in the whole study group from 8.0% to 38.9% (p < 0.001) in the use of spirometry in primary health care. This increase was significant (p < 0.001) both in the COPD group (from 32.0% to 79.6%) and "others" (from 5.6% to 32.8%) (Table 1).

Written information on smoking habits in patient records increased significantly from 16.6% of all patients in 1997 to 53.2% in 2002 (p < 0.001). Such information was obtained for 45.0% of patients in the COPD group in 1997, and significantly more often in 84.3% in 2002 (p < 0.001) (Table 1).

## Discussion

According to our study, there was a clear increase in the use of spirometry in COPD patients as well as in all patients with respiratory symptoms during the 5-year period after the introduction of the COPD programme in 1997, but also Finnish asthma programme 1994-2004 [23] and evidence-based current care guidelines for COPD in 1999 [20] may partly explain this. Written information of the smoking habits of patients was more routinely performed in 2002 compared with the year 1997 both concerning all patients with respiratory symptoms and COPD patients.

The study site was chosen because of the availability of electronic patient records, which were quite rare in Finland at the beginning of the study. It is possible that this particular health care center was more progressive than most other primary health care centers concerning implementing new guidelines. The role of specialized health care for the improvement noticed could not be assessed in this study, but it could have had a role in that. An increase in the visits due to respiratory symptoms from 1997 to 2002 was obvious and could have been partly caused by increasing public knowledge concerning COPD and respiratory diseases. Despite of that the number of COPD patients visiting primary health care was on a lower level than expected in both study years. In a recent study the prevalence of COPD was 4.3% in men and 3.1% in women in Finland [24]. Compared with this, numerous undiagnosed patients with COPD probably existed in our study population, or they did not visit primary health care. Only one diagnosis code was routinely recorded on every visit, and with this study method we were not able to find those visits including COPD as other than primary cause of visit. Some of the patients could have been followed in specialized health care; we were not able to locate them in our study. On the other hand, the Finnish diagnostic criteria for COPD [19,22] were introduced before GOLD criteria were set, and the cutoff point used for FEV1/FVC was higher than in GOLD causing a possibility of over-diagnosis of COPD.

A worrying figure was that only 8.0% of the patients had been examined by spirometry in 1997. This is in concordance with earlier studies [9,25], but far less than that (52%) in a new study from Sweden [26]. PEF follow-up was even less common. Without these two investigations, it was not possible to judge whether patients had COPD or asthma. After implementation of the national COPD programme the use of spirometry improved significantly in 2002. Despite being crucial in the diagnostic process of COPD, knowledge of deteriorated lung function affects the success of smoking cessation attemps as a part of a motivational package [27,28]. Partly due to COPD programme as well as for other reasons including a national asthma programme launched before COPD programme the training of primary health care professionals concerning COPD was improved at the same time. There were some technical improvements in the recording of spirometry data in electronic patient records in the study period, which may have partly improved the situation from 1997 to 2002.

The entire study group consisted of patients with respiratory symptoms, but in 1997, only 16.6% had smoking habits asked and recorded. Smoking is by far the biggest risk factor for COPD, and if ignored the disease will often remain undiagnosed. This situation improved greatly in 2002 when about half the patients were correctly asked about smoking and the data were recorded in patient records. During the study period, more attention was paid to anti-smoking work and corresponding legislation on the national level, which could have had a role in the improvement noticed. When patients admitted smoking, however the duration and heaviness of smoking was not always properly recorded. The total amount of cigarettes smoked in pack-years is important knowledge when estimating the harmful effects of smoking as well as when aiming to quit smoking [29].

## Conclusion

A great increase in the use of spirometry and knowledge of smoking habits of COPD patients was seen in this specific health care center. A positive impact of the national programme for detection of obstructive pulmonary disease can partly explain this, but also other improvements in the national level as well as in this particular healthcare center may have had a synergistic effect on the approach. Whether this can be generalized to other countries should be studied in the future, and it would be optimal that future national programs will include monitoring systems included in the programs.

## Competing interests

The authors declare that they have no competing interests.

## Authors' contributions

TV participated in the design of the study, collected the data and drafted the manuscript. AP conceived of the study and participated in the design of the study. KLe performed the statistical analysis. SYTJ drafted the manuscript. KLi participated in the design of the study. All authors read and approved the final manuscript.

## Pre-publication history

The pre-publication history for this paper can be accessed here:

http://www.biomedcentral.com/1471-2296/12/97/prepub
